# Antagonistic Activity against *Ascosphaera apis* and Functional Properties of *Lactobacillus kunkeei* Strains

**DOI:** 10.3390/antibiotics9050262

**Published:** 2020-05-18

**Authors:** Massimo Iorizzo, Silvia Jane Lombardi, Sonia Ganassi, Bruno Testa, Mario Ianiro, Francesco Letizia, Mariantonietta Succi, Patrizio Tremonte, Franca Vergalito, Autilia Cozzolino, Elena Sorrentino, Raffaele Coppola, Sonia Petrarca, Massimo Mancini, Antonio De Cristofaro

**Affiliations:** 1 Department of Agriculture, Environmental and Food Sciences, University of Molise, 86100 Campobasso, Italy; iorizzo@unimol.it (M.I.); silvia.lombardi@unimol.it (S.J.L.); sonia.ganassi@unimol.it (S.G.); m.ianiro@studenti.unimol.it (M.I.); francesco.letizia@unimol.it (F.L.); succi@unimol.it (M.S.); tremonte@unimol.it (P.T.); franca.vergalito@unimol.it (F.V.); a.cozzolino@studenti.unimol.it (A.C.); sorrentino@unimol.it (E.S.); coppola@unimol.it (R.C.); maxman@unimol.it (M.M.); decrist@unimol.it (A.D.C.); 2 CONAPROA, Consorzio Nazionale Produttori Apistici, 86100 Campobasso, Italy; petrarca.s@conaproa.it

**Keywords:** FLAB, chalkbrood disease, antifungal activity, *Apis mellifera*, probiotic syrup

## Abstract

Lactic acid bacteria (LAB) are an important group of honeybee gut microbiota. These bacteria are involved in food digestion, stimulate the immune system, and may antagonize undesirable microorganisms in the gastrointestinal tract. *Lactobacillus kunkeei* is a fructophilic lactic acid bacterium (FLAB) most frequently found in the gastrointestinal tracts of honeybees. *Ascosphaera apis* is an important pathogenic fungus of honeybee larvae; it can colonize the intestine, especially in conditions of nutritional or environmental stress that cause microbial dysbiosis. In this work, some functional properties of nine selected *L. kunkeei* strains were evaluated. The study focused on the antifungal activity of these strains against *A. apis* DSM 3116, using different matrices: cell lysate, broth culture, cell-free supernatant, and cell pellet. The cell lysate showed the highest antifungal activity. Moreover, the strains were shown to possess good cell-surface properties (hydrophobicity, auto-aggregation, and biofilm production) and a good resistance to high sugar concentrations. These *L. kunkeei* strains were demonstrated to be functional for use in “probiotic syrup”, useful to restore the symbiotic communities of the intestine in case of dysbiosis and to exert a prophylactic action against *A. apis.*

## 1. Introduction

### 1.1. Ascosphaera apis: The Causative Agent of Chalkbrood Disease

The eusocial nature of *Apis mellifera* has always facilitated the maintenance of a relatively constant gut microbiota. This is due to interactions among individuals in the hive environment, and mainly to trophallaxis. This term refers to the direct transfer of food or fluids from one individual to another; it is especially common among social insects such as honeybees. Along with nutrients, trophallaxis also allows the horizontal transmission of gut bacteria [1,2]. The stomachs of honeybees are full of nutrients and are therefore a favorable environment for symbiotic microorganisms. These take part in various processes, including food digestion, detoxification of harmful molecules, supply of essential nutrients, participation in the host defense system, and protection from pathogens and parasites. The gut microbiota can be influenced by various factors that can cause dysbiosis, including temperature, nutritional deficiencies, pesticides, parasites, or pathogens [3,4,5,6,7]. Gut microflora alteration may have a strong negative impact on bee immune defense, metabolism, and cognitive mechanisms [4]. The honeybee intestine, which functions in digestion and food processing, is also the site of infections caused by pathogens such as *A. apis, Nosema ceranae, Paenibacillus larvae*, and probably by many of the honeybee viruses [4,5,6,7]. Chalkbrood is a fungal disease of the honeybee caused by the opportunistic pathogen *A. apis*, belonging to the heterothallic Ascomycota. This disease is now found throughout the world, and there are indications that the incidence of chalkbrood may be on the rise [8]. The severity of the disease depends on various factors such as environmental conditions, the genetic background and general health status of the honeybees, and the virulence level of the fungal strains [8,9,10,11,12,13]. Honeybee larvae are initially infected by ingesting food contaminated by sexual spores of *A. apis*. The ascospores germinate in the anaerobic environment of the alimentary canal, and the hyphae of the mycelium subsequently penetrate the intestinal walls of the larvae and deprive them of nutrients [8,9]. After a few days, the fungus becomes visible as a fluffy white growth covering the larvae. Chalkbrood can cause a reduction in honey production and a high percentage of larvae deaths, with significant economic consequences for beekeepers [8,11,13]. The initial phase of infection can be facilitated by any nutritional or environmental stress that causes microbial dysbiosis [4,5,6].

### 1.2. Chalkbrood Disease Control by Symbiotic Bacteria

The use of intestinal microbial symbionts, such as via dietary supplementation, can improve the health status of bees and increase their productivity, stimulating the immune defenses and exerting an antimicrobial action against unwanted and pathogenic microflora [14,15,16,17,18,19,20]. The presence of lactic acid bacteria (LAB) in the honeybee digestive system has been consistently reported in literature [21], and *L. kunkeei* is a bacterium frequently present in the intestinal microbiota of honeybees. It colonizes fructose-rich niches and is actually classified as fructophilic lactic acid bacterium (FLAB) [22,23,24]. *L. kunkeei* seems to protect its niche against bacterial competitors, although the mechanism of its antimicrobial activity is still in many respects unknown [25]. Some authors have assumed that the antimicrobial mechanisms of symbiotic bacteria evolved synergistically with bees, with the purpose of defending themselves and their hosts [2,6]. Inhibition could be based on a combination of active compounds such as proteins, peptides, fatty acids, organic acids, and hydrogen peroxide [26]. Furthermore, the ability of *L. kunkeei* to colonize the intestine and form a biofilm creates a barrier against unwanted microorganisms [26,27]. While there are many scientific data on the antimicrobial activity of *L. kunkeei* towards other microorganisms, and on that of other bacterial species against *A. apis* [28,29,30,31,32,33,34], reports of antifungal activity of *L. kunkeei* against *A. apis* are still few [35]. In recent years, a number of different strategies have been developed and implemented to control chalkbrood disease. A broad range of chemotherapeutic compounds have been tested for their ability to control *A. apis* [36,37]. Unfortunately, none of the compounds tested have been able to prevent the disease [38]. Furthermore, pesticide and antifungal chemical residues in honey represent a major human health hazard [39]. There is increased interest in investigations into new and effective chalkbrood control methods. The use of natural compounds for the disease control also represents an alternative. Some essential oils and other botanical extracts from plants, herbs, and spices exhibit antimicrobial activity against *A. apis* [40,41,42,43,44,45]. This antimicrobial activity is mainly due to the presence of phenolic and terpenoid compounds, which have well-known antimicrobial activity. However, the effect that these substances may have on bees’ intestinal microflora and symbiotic LAB is not wholly known [46,47,48,49,50,51,52]. According to these considerations, the use of symbiotic FLAB in the prevention and biocontrol of honeybee pathogenic microorganisms, including chalkbrood disease, offers interesting possibilities [18]. The use of symbiotic bacteria, unlike synthetic or natural chemical compounds, does not adversely affect the balance of gut microbiota or impact honeybee health [4,7,21,33].

In this research, the antifungal activity of nine *L. kunkeei* strains against *A. apis* was evaluated. Moreover, further functional characteristics (auto-aggregation, biofilm production, hydrophobicity, and osmotic tolerance) were also assayed to assess suitability for use in a “probiotic syrup” to enhance the honeybee diet.

## 2. Results

### 2.1. Screening of Bacteria for Antifungal Activity

In the preliminary antifungal tests, all 85 *L. kunkeei* strains showed antifungal activity, but with different intensity: 8 strains had low intensity, 54 strains had medium intensity, and 23 strains had high intensity (Supplementary Materials Table S1).

Among the 23 strains that showed high antifungal activity, the strains K7, K18, K34, K40, K41, K45, K55, K64, and K112 caused 100% inhibition and were selected for further biological control tests against *A. apis* DSM 3116 and subsequent analyses. 

### 2.2. Determination of Inhibitory Activity

**Figure 1:**
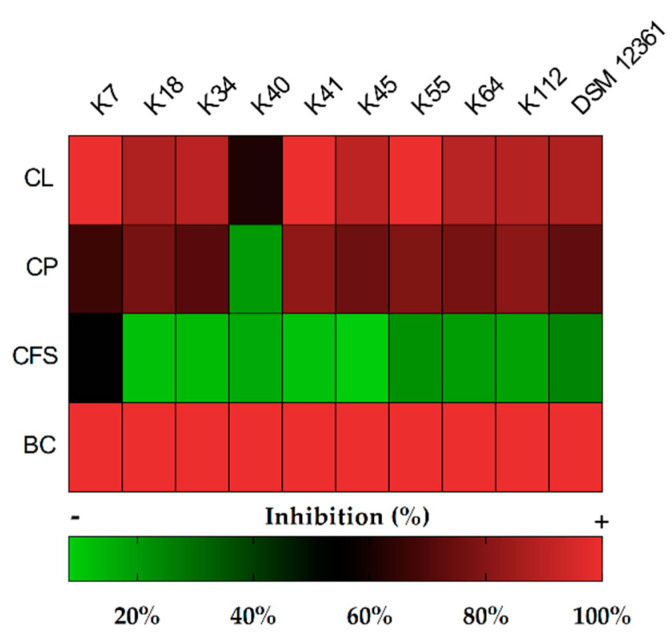
Heatmap of inhibition (%) against *Ascosphaera apis* DSM 3116 (radial growth) on Malt Extract Agar (MEA) plates after 6 days, using culture broth (CB), cell pellets (CPs), cell-free supernatants (CFSs), and cell lysates (CLs) of the *Lactobacillus kunkeei* strains.

Figure 1 presents a heatmap of the inhibitory activity against *A. apis* DSM 3116 of the various matrices obtained from bacterial cell cultures. The broth cultures (BCs) of the nine selected strains confirmed total inhibition against the fungus, as already highlighted in the previous screening test. 

In tests where the other matrices Cell Lysate (CL), Cell Pellet (CP) and Cell-Free Supernatant (CFS) were used, there was variability with often significant differences. Numeric data are shown in Supplementary Materials Table S2.

**Figure 2:**
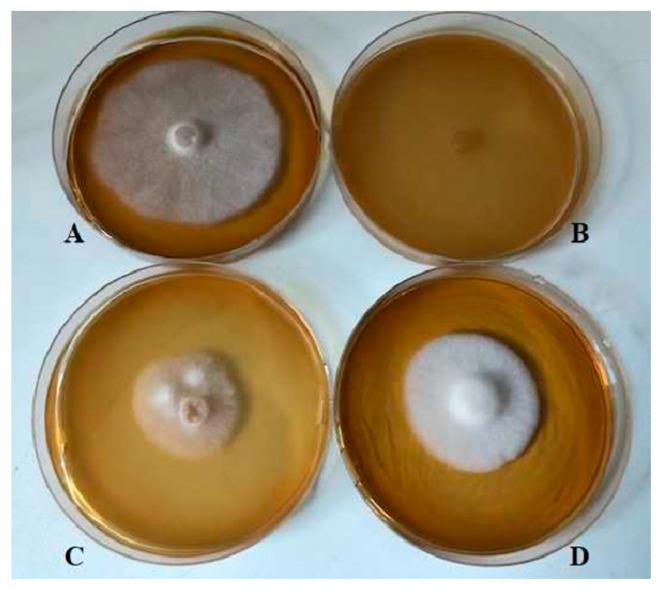
Inhibitory activity after 6 days on MEA agar plates of *L. kunkeei* K7 against *A. apis* DSM 3116. (**A**): *A. apis*; (**B**): *A. apis* + CL (cell lysate); (**C**): *A. apis* + CP (cell pellet); (**D**): *A. apis* + CFS (cell-free supernatant).

The CLs inhibited the fungus more than other matrices; in particular, the K7, K41, K45, and K55 *L. kunkeei* strains caused inhibition of more than 90%. The CPs showed a high inhibitory activity ranging from 60% to 80%, with the exception of the *L. kunkeei* K40 strain (19.8%). The CFSs showed overall less inhibitory activity, between 8% and 24%, with the exception of the CFS of *L. kunkeei* K7, which caused 53.8% inhibition (Figure 2).

### 2.3. Hydrophobicity and Auto-Aggregation

**Figure 3:**
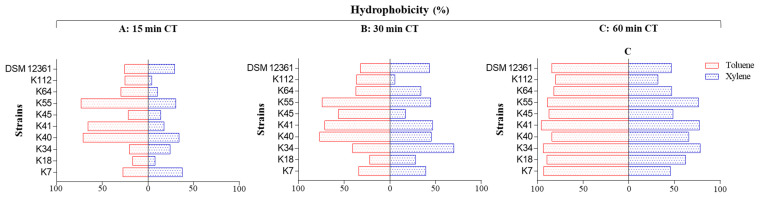
Adhesion of the *L. kunkeei* strains to toluene and xylene (expressed as hydrophobicity %) measured using bacterial ability to adhere to hydrocarbons (BATH) test after different contact times (CTs). (**A**): 15 min; (**B**): 30 min; (**C**): 60 min.

This section reports the results of the tests of hydrocarbon adhesion and the auto-aggregation capacity of the selected *L. kunkeei* strains. The results are displayed graphically in Figure 3 and numerically in Supplementary Materials Table S3.

The hydrophobicity was highly variable depending on the strain and the hydrocarbon used, with significant differences in almost all cases. All the strains showed high adherence to toluene, while only three strains (K34, K41, and K55) showed high adherence to xylene. The adhesion to the two hydrocarbons gradually increased during the 60 min of tests, and the percentage of hydrophobicity obtained with toluene was significantly higher than that with xylene. In the test with toluene, three strains (K7, K34, and K41) showed an affinity greater than 90% after 60 min; the other strains showed a high hydrophobicity between 80% and 90%.

In the test with xylene, three strains (K34, K41, and K55) showed high hydrophobicity greater than 70%; the K7, K18, K40, K45, K64, and DSM 12361 strains showed moderate hydrophobicity between 45% and 66%; K112 alone showed a low affinity (31.91%).

The results of the auto-aggregation test are shown in Table 1. All nine strains demonstrated an ability of auto-aggregation that progressively and significantly increased over time.

The data showed significant differences among the various strains after 1, 2 and 5 h. After 24 h, there was less variability; in fact, the auto-aggregation capacity of the K7, K40, K64, K112, and DSM 12361 strains, ranging between 53.42% and 56.52%, was not significantly different.

### 2.4. Biofilm Production

**Figure 4:**
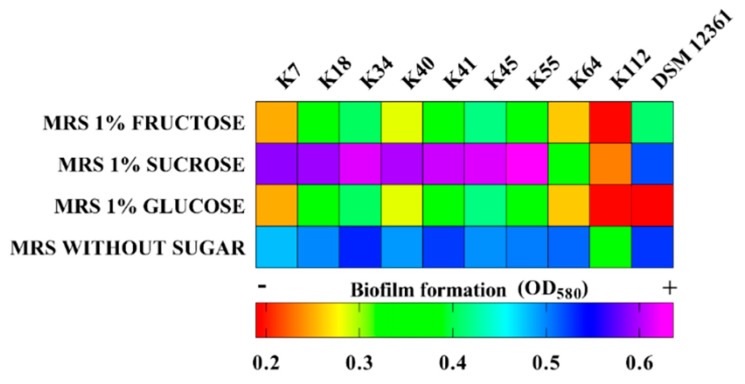
Biofilm formation in vitro of the *L. kunkeei* strains, expressed as Optical Density (OD) value at 580 nm.

Figure 4 shows the heatmap of biofilm production in MRS broth without sugar or supplemented with 1% glucose, 1% fructose, or 1% sucrose. The amount of biofilm was assessed by measuring the optical density (OD), and numerical data are provided in Supplementary Materials Table S4. All the *L. kunkeei* strains tested were able to produce biofilms, but in different amounts depending on the presence and type of added sugar.

With the addition of 1% of glucose or 1% fructose, the biofilm production for each bacterial strain was similar, but among the various strains, the OD values were significantly different.

In MRS without sugar, five strains (K34, K41, K55, K64, and DSM 12361) produced the largest amounts of biofilm, to a significantly different degree than the other strains. With the addition of sucrose, the largest amounts of biofilms were produced by K7, K18, K34, K40, K41, K45, and K55 *L. kunkeei* strains, with the differences among them not statistically significant.

### 2.5. Bacterial Survival in Sugar Syrup

The results of strains’ survival in sugar syrup are shown in Table 2. In Test A (40% glucose + 20% fructose, pH 4.2), after 24 h of incubation, all the strains maintained a high viable cell density between 6.90 and 8.10 log CFU/mL; after 48 h, although with significant differences, the all strains maintained a good cell density ranging between 4.80 and 7.51 log CFU/mL.

In Test B (40% glucose + 30% fructose at pH 4.2), after 24 h, the bacteria, except the strains DSM 12361 and K112, showed concentrations of viable cells greater than 6.0 log CFU/mL; after 48 h, only K18 and K55 maintained a good viable cell density (>5.0 log CFU/mL), with results significantly different from the other strains. The lowest concentration was that of the DSM 12361 strain (2.91 log CFU/mL). In Test C (50% of sucrose at pH 4.2), after 24 h, the concentration of viable cells for all strains was between 7.10 and 8.50 log CFU/mL, while after 48 h, although with significant differences, all strains maintained a good viable cell density between 5.94 and 7.94 log CFU/mL.

## 3. Discussion

### 3.1. Antifungal Activity

In the inhibition test against *A. apis*, all nine *L. kunkeei* strains showed strong antifungal activity. Complete inhibition occurred with the use of BC, which was most likely due to the interaction of several factors. The inhibitory effects observed when using CP and CL were stronger than those obtained with the CFS. Our results suggest that the antimicrobial action of LAB is often due to a complex interaction among different compounds (e.g., organic acids, fatty acids, proteinaceous compounds, phenolic acids, hydrogen peroxide, reuterin) contained in the different matrices used, as highlighted in other research [53,54,55,56]. The action in CP could be based on either nutritional competition or compounds linked to their walls. The major inhibitory effect after cell lysis is probably due to the release of further antimicrobial compounds from the cell wall or from the cytoplasm. In general, LAB can produce extracellular proteins such as bacteriocins, molecular chaperones, enzymes, and lipoproteins [57]. Glycolytic and ribosomal proteins generally contained in the cytoplasm can be found on the bacterial cell surface. It is hypothesized that these proteins, once they are localized on the surface, could develop different functions such as biofilm production and antimicrobial activity [21,58,59]. The *L. kunkeei* strains tested in our experiments were shown to possess substances biologically active against *A. apis*. Our results confirmed a potentially antagonist role of *L. kunkeei* against pathogenic microorganisms that use the digestive channels of bees as a site of infection [22,60,61].

### 3.2. Cell-Surface Properties

The ability of probiotic bacteria to adhere to intestinal epithelial cells involves various surface properties, including hydrophobicity and auto-aggregation. These characteristics are an important prerequisite for colonization of the host intestinal tract [62,63,64,65,66,67]. The probiotic bacteria are linked to the receptors of intestinal mucosa, thus preventing the adherence of pathogenic microorganisms which are subsequently eliminated from the intestine [62,63]. The quality of adherence, usually strain-specific, can also cause increased persistence in the gastrointestinal tract. The adherence property is most likely due to complex interactions between positive and negative charges between hydrophobic and hydrophilic components of the bacterial surface [64]. As reported in previous studies, hydrophobicity, together with auto-aggregation, is considered an important bacterial surface feature [65,66], and correlations between them may be observed [67]. In our study, the hydrophobicity was evaluated by BATH method using two different hydrocarbons, xylene and toluene [67], and classified into three groups: low (0% to 35%), moderate (36% to 70%), and high hydrophobicity (71% to 100%) [68]. The BATH assay showed significant variations depending on the hydrocarbon used. The nine *L. kunkeei* strains all showed an enhanced adherence capacity to toluene than xylene. In previous studies, it has been reported that the viscosity of the hydrocarbon or the size of droplets formed during mixing may determine this difference [69].

In the future, it will be necessary to perform this assay with cell lines to confirm the adhesion of the tested strains to epithelial cells.

Auto-aggregation is a characteristic that promotes the stability of microbial strains in the gastrointestinal tract (GIT). This phenotypic characteristic, as several studies have shown, can be constitutive or induced by nutritional and environmental stress conditions (pH, temperature, presence of competing microorganisms, etc.) [63,64,65,66,67,68,69,70]. Our results showed that the nine *L. kunkeei* strains had high values of auto-aggregation, in line with previous research [71]. The bacterial auto-aggregation capacity is related to the building of a biofilm. Some microorganisms have the ability to form biofilms to adhere to surfaces. Bacterial biofilm production is a highly complex process depending on the expression of specific genes, and this process is a means by which to adapt to new nutritional and environmental conditions [70]. Several studies have reported that in this phase of adaptation, bacteria produce extracellular polymeric substances (EPS), especially exopolysaccharides and proteinaceous compounds, that may be constituents of the biofilm and could exert an antimicrobial action [72,73,74,75,76].

In our study, all the *L. kunkeei* strains were shown to be able to produce biofilm, with different intensities.

In this regard, the best results were obtained with the use of 1% sucrose and without adding sugar. According to some authors, it would seem that in the presence of a nutritional stress, the strains used the biofilm as a defense mechanism [21,77,78,79]. Thanks to its ability to produce biofilms, *L. kunkeei* persists in the intestine where there is an extensive flow of sugars, enzymes, and water, and the constant invasion of foreign microbes following the ingestion of flower nectar during foraging. As highlighted in other research, the biofilms formed by some *L. kunkeei* strains favor their persistence in the bee intestine and increase their inhibition power against undesirable microorganisms [15,18,20,79].

### 3.3. Survival in Sugar Syrups

In our experimental studies, we evaluated the capacity of *L. kunkeei* strains to tolerate a high concentration of sugars to verify their functionality as probiotics in a sugar syrup used as additional nutrition for bees. The results proved that all nine strains, in all combinations, had a good osmotic tolerance, in agreement with a previous study [22]. This feature would ensure a high bacterial vitality if *L. kunkeei* were added to sugar syrups used as additional food in hives. Moreover, honeybees are attracted by highly concentrated sugar syrup; this behavior becomes important when finding a compromise between maximum attractiveness for bees and survival of lactic bacteria.

### 3.4. Perspectives

Several studies have shown that the use of probiotic bacteria in honeybee diet may have several positive effects, including increasing the immune defenses of bees, strengthening or rebalancing the intestinal microflora in case of dysbiosis, improving bee productivity, and strengthening defense systems against pathogenic microorganisms [3,23,30,31,32,33,34,35,80,81,82,83,84,85].

The novelty of our work was to select nine symbiotic *L. kunkeei* strains for use in a honeybee diet for prophylactic and therapeutic purposes against *A. apis*. The selected *L. kunkeei* strains showed functional properties (cell surface properties, antifungal activity, and osmotic tolerance) for use as probiotics in sugar syrups to be used in the supplemental feeding of honeybees.

## 4. Materials and Methods

### 4.1. Microbial Cultures

For this study, 85 selected *L. kunkeei* strains were used, isolated from bee bread, honey stomach, and honeybee guts of *Apis mellifera* L. (Supplementary Materials Table S1). These bacteria belong to the Di.A.A.A (Department of Agricultural, Environmental and Food Sciences) collection of University of Molise [85].

*A. apis* DSM 3116 and *L. kunkeei* DSM 12361, belonging to the DSMZ collection (German Collection of Microorganism and Cell Cultures GmbH), were used as reference cultures. We used the DSM 12361 strain as a reference because it has demonstrated a capacity in previous research to produce EPS [76,77,78].

### 4.2. Screening of Bacteria for Antifungal Activity

The antifungal activity screening for the 85 *L. kunkeei* strains was performed using the overlay method as described by Magnusson et al. [86], with some modification. The strains were cultivated in MRS broth (Oxoid Ltd., Hampshire, UK) at 37 °C for 12 h, after which 10 μL of the bacterial culture (10^8^ CFU/mL) was spotted onto the surface of MRS agar plates and plates were incubated at 37 °C for 24 h. Fungal culture of *A. apis* DSM 3116 was obtained by growing on a MEA plate (Oxoid Ltd., Hampshire, UK) aerobically at 37 °C for 5 days. After that, a mycelium disc of 6 mm diameter was taken, dissolved in physiological solution (0.9% NaCl), and vortexed for 5 min; subsequently, 1 mL of the fungal suspension was inoculated into a tube containing 9 mL of MEA soft agar (0.05% malt extract and 0.7% agar) that was overlaid in the Petri dishes with the various bacterial strains. A plate containing MEA with fungal suspension and without bacteria was used as a control. After 72 h of incubation at 37 °C, the inhibition activity was measured as the diameter (mm) of the clear zone around the bacterial spot; the different activity levels of the bacteria were classified as follows: 0–30 mm: low activity; 30–60 mm: medium activity; >60 mm: high activity.

### 4.3. Determination of Inhibitory Activity

The inhibitory activity was performed using the following matrices: BC, CP, CFS, and CL. In order to obtain the fractions, every single strain was cultivated in MRS broth and incubated at 37 °C for 12 h, reaching a cell concentration of 10^8^ CFU/mL. This culture without any further treatment was the BC matrix. Five milliliters of the bacterial culture were centrifuged at 8000 rpm for 15 min at 4 °C. After centrifugation, the supernatant was sterilized by filtration (0.22 μm pore size cellulose acetate filter), and this fraction represented the CFS. The remaining pellet was washed and resuspended in 5 mL of physiological solution, which was the CP fraction. In order to obtain the CL fraction, 5 mL of the BC was centrifuged and the pellet washed, resuspended in 5 mL of physiological solution, and sonicated (20 kHz for 30 min a 45 °C) to promote cellular lysis. A volume of 5mL of each matrix (BC, CP, CFS, and CL), was added to 15 mL of MEA; the preparation was poured into 90 mm Petri dishes. After solidification, a mycelium disc (6 mm diameter) of *A. apis* (DSM 3116) was placed in the middle of each Petri dish and incubated at 37 °C in aerobic conditions. The antifungal activity was evaluated by measuring the hyphal radial growth (cm diameter) after 6 days of incubation and expressed as percentage of inhibition using the following formula: % I = [1 − (Ds/Dc)] × 100, where Ds was the hyphal diameter of the sample and Dc was the hyphal diameter of the control (MEA only with fungus).

### 4.4. Hydrophobicity Assay

The determination of cell-surface hydrophobicity was evaluated on *L. kunkeei* strains basing on the bacterial ability to adhere to hydrocarbons (BATH), according to the procedure described by Cozzolino et al. [87], using xylene and toluene as solvents. The bacteria were grown overnight at 37 °C in MRS broth. Cultures were collected by centrifugation (8000 rpm for 10 min at 4 °C) during the logarithmic growth phase, washed twice and resuspended in physiological solution to an optical density of approx. 0.5 (A_580_), in order to standardize the bacterial concentration at 10^8^ CFU/mL. An equal volume of hydrocarbon (xylene or toluene) was then added to the bacterial suspension, mixed (in a vortex-type mixer) for 5 min and incubated at 37 °C. The aqueous phase was carefully removed after 15, 30, and 60 min of incubation at room temperature and the absorbance was measured at 580 nm using a spectrophotometer (PerkinElmer 1420 Multilabel Counter). Hydrophobicity was calculated as the percentage decrease in OD of the initial bacterial suspension and was expressed using the following formula: % Hydrophobicity = (OD_0_ − ODt/OD_0_) × 100, where ODt represents the absorbance value after extraction with hydrocarbons (15, 30, and 60 min) and OD_0_ represents the absorbance value before extraction with hydrocarbons. The *L. kunkeei* strains were classified into three roups on the basis of their affinity to hydrocarbons: low (0% to 35%), moderate (36% to 70%), and high hydrophobicity (71% to 100%) [68].

### 4.5. Auto-Aggregation

The auto-aggregation assay was performed according to Collado et al. [67]. The bacterial suspensions were prepared as described for BATH test. Auto-aggregation was measured at 1, 2, 5, and 24 h of incubation at 37 °C, after which the OD at 580nm of the upper suspension was measured using a spectrophotometer (PerkinElmer 1420 Multilabel Counter). The percentage of auto-aggregation was calculated using the following formula: Auto-aggregation % (A) = (1 − ODt/OD_0_) × 100 where OD_0_ is the absorbance at time 0 and ODt is the absorbance detected after 1, 2, 5, and 24 h [64].

### 4.6. Biofilm Production

Biofilm production was evaluated as described by Cozzolino et al. [87] with some modifications. *L. kunkeei* strains were grown overnight at 37 °C in MRS broth. The bacterial cells were harvested by centrifugation at 8000 rpm for 10 min at 4 °C, washed twice with PBS (Sigma-Aldrich), and resuspended in MRS broth without glucose (Liofilchem, Italy) and in MRS broth supplemented with 1% glucose, 1% fructose, or 1% sucrose. Three aliquots of 200 μL of each bacterial suspension were added to a 96 well polystyrene microtiter plate. Negative controls were constituted by wells filled with uninoculated culture media. Microtiter plates were incubated for 24 h at 37 °C. The medium was removed from each well and plates were washed three times with a sterile physiological solution to remove unattached cells. The remaining attached cells were fixed with 200 μL of 99% methanol (Sigma–Aldrich) per well. After 15 min, wells were emptied and left to dry. Wells were then stained for 5 min with 200 μL of 2% crystal violet (Liofilchem, Italy) per well. The excess stain was removed by washing three times with a sterile saline solution. After the plates were air-dried, the adherent cells were resuspended in 160 μL of 33% (*v*/*v*) glacial acetic acid (Sigma–Aldrich). The absorbance values at 580 nm, measured using an automated Multilabel Counter (PerkinElmer 1420), represent the biofilm formation capacity.

### 4.7. Bacterial Survival in Sugar Syrup

The *L. kunkeei* strains were grown overnight in MRS broth at 37 °C. After that, cells were harvested by centrifugation at 8000 rpm for 10 min at 4 °C. The fresh pellets were washed twice with physiological solution and inoculated into the sugar syrup in order to measure an initial concentration of 10^8^ CFU/mL. The experimental conditions were as follows: Test A: sugar syrup constituted of 40% glucose + 20% fructose in distilled water at pH 4.2; Test B: sugar syrup constituted of 40% glucose + 30% fructose in distilled water at pH 4.2; Test C: sugar syrup constituted of 50% sucrose in distilled water at pH 4.2. The sugar syrup was acidified using HCl 1N and sterilized by filtration (0.22 μm pore size cellulose acetate filter). The experiments were performed at 20 °C. The cell viability of the bacteria was determined at 0, 24, and 48 h by plating on MRS agar (37 °C for 72 h in anaerobic conditions).

### 4.8. Statistical Analysis

All data are expressed as mean ± standard deviation (S.D.) of three independent experiments. Statistical analysis was performed via an analysis of variance (ANOVA) followed by the Tukey’s multiple comparison. Statistical significance was attributed to *p*-values < 0.05. The software SPSS (IBM SPSS Statistics 21) was used for the analysis.

## 5. Conclusions

The results showed the capacity of selected *L. kunkeei* strains to inhibit *A. apis* and highlighted their important properties, such as ability to form biofilms, high auto-aggregation, and hydrophobicity, all prerequisites for candidacy as probiotic microorganisms in a honeybee diet. In addition, the selected *L. kunkeei* strains showed high osmotic tolerance, a functional requirement for the probiotication of sugar syrups to restore or strengthen the symbiotic communities in honeybee guts in case of microbial dysbiosis.

Future research will be conducted in vivo/in situ on the antimicrobic activity of these bacteria and the effects on honeybee health and productivity.

## Figures and Tables

**Table 1:** Auto-aggregation (%) of the *L. kunkeei* strains after 1, 2, 5, and 24 h of incubation at 37 °C. Results are shown as mean ± standard deviation (n = 3). Different lowercase letters (a-d) indicate significant differences by column, and different uppercase letters (A-I) in each row indicate significant differences by row (*p* < 0.05).

Time (Hours)	Auto-Aggregation (%)									
	K7	K18	K34	K40	K41	K45	K55	K64	K112	DSM 12361
1	11.10 ± 0.88^Ga^	4.49 ± 0.14^Ca^	4.51 ± 0.10^Ca^	15.41 ± 0.54^Ha^	3.25 ± 0.12^Ba^	0.76 ± 0.02^Aa^	5.55 ± 0.22^Da^	6.76 ± 0.50^Ea^	9.48 ± 0.16^Fa^	9.32 ± 0.20^Fa^
2	18.40 ± 1.18^Fb^	8.62 ± 0.36^Cb^	10.94 ± 0.89^Db^	17.40 ± 1.08^Fb^	7.56 ± 0.32^Bb^	2.11 ± 0.10^Ab^	10.43 ± 0.27^Db^	10.61 ± 0.35^Db^	16.10 ± 0.96^Fb^	13.66 ± 0.35^Eb^
5	22.71 ± 1.00^Fc^	12.41 ± 0.16^Cc^	11.31 ± 0.34^Bb^	24.94 ± 0.04^Hc^	25.20 ± 0.19^Ic^	6.23 ± 0.59^Ac^	14.82 ± 0.98^Dc^	15.27 ± 0.12^Dc^	23.42 ± 0.31^Gc^	19.70 ± 0.65^Ec^
24	53.42 ± 1.21^Bd^	62.23 ± 1.32^Dd^	65.62 ± 1.37^Ec^	56.52 ± 1.95^Cd^	68.10 ± 1.71^Fd^	41.81 ± 0.42^Ad^	62.30 ± 0.99^Dd^	56.12 ± 2.39^Cd^	55.80 ± 0.94^Cd^	55.42 ± 3.21^Cd^

**Table 2:** Survival of the *L. kunkeei* strains in different sugar syrups after 24 and 48 h of incubation at 20 °C. Results are shown as mean ± standard deviation (n = 3). For every sugar syrup, different lowercase letters (a-c) indicate significant differences in columns, and different uppercase letters (A-I) indicate significant differences in rows (*p* < 0.05).

Time (Hours)	Sugar Syrup Composition	Survival (log CFU/mL) of *L. kunkeei* Strains									
		K7	K18	K34	K40	K41	K45	K55	K64	K112	DSM 12361
T_0_	40% glucose 20% fructose	8.41 ± 0.02^Bc^	8.38 ± 0.04^Bc^	8.44 ± 0.06^Bc^	8.23 ± 0.03^Ac^	8.19 ± 0.04^Ac^	8.17 ± 0.02^Ac^	8.26 ± 0.05^Ac^	8.24 ± 0.01^Ac^	8.36 ± 0.02^Bc^	8.40 ± 0.04^Bc^
T_24_		7.89 ± 0.01^Eb^	8.10 ± 0.01^Gb^	7.60 ± 0.07^Cb^	7.12 ± 0.05^Bb^	7.20 ± 0.04^Bb^	7.80 ± 0.06^Db^	7.18 ± 0.08^Bb^	6.90 ± 0.04^Ab^	8.01 ± 0.01^Fb^	6.90 ± 0.02^Ab^
T_48_		6.62 ± 0.03^Fa^	7.51 ± 0.02^Ha^	4.90 ± 0.02^Ba^	5.84 ± 0.05^Ca^	6.29 ± 0.01^Da^	6.47 ± 0.02^Ea^	5.84 ± 0.01^Ca^	5.90 ± 0.02^Ca^	6.84 ± 0.03^Ga^	4.80 ± 0.04^Aa^
T_0_	40% glucose 30% fructose	8.66 ± 0.04^Ec^	8.50 ± 0.03^Dc^	8.43 ± 0.05^Dc^	8.06 ± 0.08^Ac^	8.46 ± 0.08^Dc^	8.43 ± 0.02^Dc^	8.25 ± 0.04^Bc^	8.23 ± 0.05^Bc^	8.30 ± 0.02^Cc^	8.45 ± 0.06^Dc^
T_24_		6.23 ± 0.02^Db^	7.12 ± 0.05^Gb^	6.24 ± 0.04^Db^	6.90 ± 0.02^Fb^	6.84 ± 0.01^Eb^	6.11 ± 0.05^Cb^	6.12 ± 0.02^Cb^	6.24 ± 0.06^Db^	5.97 ± 0.02^Bb^	4.97 ± 0.07^Ab^
T_48_		4.54 ± 0.04^Da^	5.75 ± 0.05^Ha^	4.61 ± 0.01^Ea^	4.01 ± 0.02^Ba^	4.98 ± 0.03^Ga^	4.73 ± 0.03^Fa^	5.59 ± 0.01^Ia^	4.94 ± 0.04^Ga^	4.20 ± 0.02^Ca^	2.91 ± 0.01^Aa^
T_0_	50% sucrose	8.20 ± 0.04^Bc^	8.10 ± 0.01^Ac^	8.50 ± 0.03^Fc^	8.40 ± 0.06^Dc^	8.49 ± 0.07^Ec^	8.23 ± 0.01^Bc^	8.10 ± 0.04^Ac^	8.53 ± 0.07^Fc^	8.50 ± 0.06^Fc^	8.30 ± 0.04^Cc^
T_24_		7.20 ± 0.04^Bb^	8.20 ± 0.03^Gb^	7.90 ± 0.02^Fb^	7.79 ± 0.06^Eb^	8.50 ± 0.01^Hb^	7.60 ± 0.02^Db^	7.10 ± 0.01^Ab^	7.60 ± 0.05^Db^	7.50 ± 0.07^Cb^	7.20 ± 0.06^Bb^
T_48_		7.00 ± 0.05^Da^	7.94 ± 0.01^Fa^	5.94 ± 0.02^Aa^	6.85 ± 0.01^Ca^	7.30 ± 0.05^Ea^	6.89 ± 0.02^Ca^	6.85 ± 0.01^Ca^	6.90 ± 0.05^Ca^	6.90 ± 0.06^Ca^	6.50 ± 0.06^Ba^

## Supplementary Materials

The following are available online at https://www.mdpi.com/2079-6382/9/5/262/s1. Table S1: L. *kunkeei* strains collection; Table S2: Inhibition; Table S3: Hydrophobicity; Table S4: Biofilm production.
