# Supplementary material for: Antagonistic Activity against Ascosphaera apis and Functional Properties of Lactobacillus kunkeei Strains
Source: Antibiotics (Basel). 2020 May 18;9(5):262. doi: 10.3390/antibiotics9050262 (PMC7277644; doi:10.3390/antibiotics9050262)
Supplement: Supplementary file 1 [file antibiotics-09-00262-s001.zip › Supplementary material/Table S2 .pdf]

	Biofilm formation (OD_{580})									
**Medium condition**	**K7**	**K18**	**K34**	**K40**	**K41**	**K45**	**K55**	**K64**	**K112**	**DSM 12361**
MRS 1% FRUCTOSE	0.248±0.015	0.377±0.018	0.404±0.016	0.285±0.007	0.361±0.017	0.414±0.014	0.378±0.016	0.258±0.011	0.193±0.015	0.408±0.016
MSR 1% SUCROSE	0.589±0.020	0.593±0.007	0.613±0.030	0.599±0.014	0.607±0.011	0.613±0.008	0.636±0.008	0.360±0.009	0.233±0.005	0.519±0.009
MRS WITHOUT SUGAR	0.480±0.018	0.498±0.020	0.533±0.027	0.493±0.005	0.525±0.020	0.496±0.007	0.503±0.010	0.510±0.021	0.320±0.016	0.527±0.011
MRS 1% GLUCOSE	0.248±0.010	0.377±0.011	0.404±0.025	0.285±0.018	0.361±0.023	0.414±0.023	0.378±0.015	0.258±0.008	0.193±0.007	0.188±0.007
