# Supplementary material for: Antagonistic Activity against Ascosphaera apis and Functional Properties of Lactobacillus kunkeei Strains
Source: Antibiotics (Basel). 2020 May 18;9(5):262. doi: 10.3390/antibiotics9050262 (PMC7277644; doi:10.3390/antibiotics9050262)
Supplement: Supplementary file 1 [file antibiotics-09-00262-s001.zip › Supplementary material/Captions.docx]

**Table S1**. Adhesion of the nine *L. kunkeei* selected strains and *L. kunkeei* DSM 12361 to hydrocarbon (expressed as Hydrophobicity %) measured using the BATH test after 15, 30 and 60 min (contact time). The values represent the average (± SD) of three biological replicates.

**Table S2**. Biofilm formation (expressed as OD value at 580nm) of *L. kunkeei* strains in MRS with glucose, fructose, sucrose or without sugar, after incubation at 37 C for 24 h. The values represent the average (± SD) of three biological replicates.
