# Supplementary material for: Antagonistic Activity against Ascosphaera apis and Functional Properties of Lactobacillus kunkeei Strains
Source: Antibiotics (Basel). 2020 May 18;9(5):262. doi: 10.3390/antibiotics9050262 (PMC7277644; doi:10.3390/antibiotics9050262)
Supplement: Supplementary file 1 [file antibiotics-09-00262-s001.zip › Supplementary material/Table S1.pdf]

	Contact time (min)	Hydrophobicity (%)									
		**K7**	**K18**	**K34**	**K40**	**K41**	**K45**	**K55**	**K64**	**K112**	**DSM 12361**
Toluene	15	27.30±0.51	16.52±0.51	20.31±0.35	70.96±1.81	65.46±1.41	21.68±0.44	73.25±1.85	29.57±0.31	25.35±0.41	25.54±0.40
	30	34.00±0.61	21.81±0.94	40.80±1.15	77.10±1.92	71.20±1.16	56.44±2.28	74.11±1.01	37.10±1.55	36.63±0.70	32.23±1.55
	60	93.40±1.10	89.70±1.40	93.60±1.83	84.12±0.86	95.91±0.94	87.40±1.97	89.20±0.53	82.01±1.90	80.30±1.67	84.34±0.86
Xylene	15	37.75±2.44	7.61±0.59	24.3±1.46	34.09±0.99	17.69±1.22	14.08±0.63	30.76±1.51	10.54±0.62	4.25±0.27	29.34±1.41
	30	39.61±1.34	28.56±0.75	69.99±3.53	45.54±1.24	46.98±1.66	17.16±0.85	44.99±0.81	34.16±0.88	5.73±0.37	43.44±0.54
	60	45.89±3.87	62.34±1.94	78.17±4.17	65.64±4.38	77.37±2.97	48.53±1.08	76.64±2.37	46.81±1.04	31.91±0.94	46.77±0.82
