# Supplementary material for: Antagonistic Activity against Ascosphaera apis and Functional Properties of Lactobacillus kunkeei Strains
Source: Antibiotics (Basel). 2020 May 18;9(5):262. doi: 10.3390/antibiotics9050262 (PMC7277644; doi:10.3390/antibiotics9050262)
Supplement: Supplementary file 1 [file antibiotics-09-00262-s001.zip › Supplementary material/Table S2 .docx]

|  |  |  |  | **Biofilm formation (OD_580_)** | | |  |  |  |  |
| --- | --- | --- | --- | --- | --- | --- | --- | --- | --- | --- |
| **Medium condition** | **K7** | **K18** | **K34** | **K40** | **K41** | **K45** | **K55** | **K64** | **K112** | **DSM 12361** |
| MRS 1% FRUCTOSE | 0.248±0.015 | 0.377±0.018 | 0.404±0.016 | 0.285±0.007 | 0.361±0.017 | 0.414±0.014 | 0.378±0.016 | 0.258±0.011 | 0.193±0.015 | 0.408±0.016 |
| MSR 1% SUCROSE | 0.589±0.020 | 0.593±0.007 | 0.613±0.030 | 0.599±0.014 | 0.607±0.011 | 0.613±0.008 | 0.636±0.008 | 0.360±0.009 | 0.233±0.005 | 0.519±0.009 |
| MRS WITHOUT SUGAR | 0.480±0.018 | 0.498±0.020 | 0.533±0.027 | 0.493±0.005 | 0.525±0.020 | 0.496±0.007 | 0.503±0.010 | 0.510±0.021 | 0.320±0.016 | 0.527±0.011 |
| MRS 1% GLUCOSE | 0.248±0.010 | 0.377±0.011 | 0.404±0.025 | 0.285±0.018 | 0.361±0.023 | 0.414±0.023 | 0.378±0.015 | 0.258±0.008 | 0.193±0.007 | 0.188±0.007 |
