# Supplementary material for: Antagonistic Activity against Ascosphaera apis and Functional Properties of Lactobacillus kunkeei Strains
Source: Antibiotics (Basel). 2020 May 18;9(5):262. doi: 10.3390/antibiotics9050262 (PMC7277644; doi:10.3390/antibiotics9050262)
Supplement: Supplementary file 1 [file antibiotics-09-00262-s001.zip › Supplementary material/Table S1.docx]

|  | **Contact time (min)** | **Hydrophobicity (%)** | | | | | | | | |  |
| --- | --- | --- | --- | --- | --- | --- | --- | --- | --- | --- | --- |
|  |  | **K7** | **K18** | **K34** | **K40** | **K41** | **K45** | **K55** | **K64** | **K112** | **DSM 12361** |
| Toluene | 15 | 27.30±0.51 | 16.52±0.51 | 20.31±0.35 | 70.96±1.81 | 65.46±1.41 | 21.68±0.44 | 73.25±1.85 | 29.57±0.31 | 25.35±0.41 | 25.54±0.40 |
|  | 30 | 34.00±0.61 | 21.81±0.94 | 40.80±1.15 | 77.10±1.92 | 71.20±1.16 | 56.44±2.28 | 74.11±1.01 | 37.10±1.55 | 36.63±0.70 | 32.23±1.55 |
|  | 60 | 93.40±1.10 | 89.70±1.40 | 93.60±1.83 | 84.12±0.86 | 95.91±0.94 | 87.40±1.97 | 89.20±0.53 | 82.01±1.90 | 80.30±1.67 | 84.34±0.86 |
| Xylene | 15 | 37.75±2.44 | 7.61±0.59 | 24.3±1.46 | 34.09±0.99 | 17.69±1.22 | 14.08±0.63 | 30.76±1.51 | 10.54±0.62 | 4.25±0.27 | 29.34±1.41 |
|  | 30 | 39.61±1.34 | 28.56±0.75 | 69.99±3.53 | 45.54±1.24 | 46.98±1.66 | 17.16±0.85 | 44.99±0.81 | 34.16±0.88 | 5.73±0.37 | 43.44±0.54 |
|  | 60 | 45.89±3.87 | 62.34±1.94 | 78.17±4.17 | 65.64±4.38 | 77.37±2.97 | 48.53±1.08 | 76.64±2.37 | 46.81±1.04 | 31.91±0.94 | 46.77±0.82 |
